# Accurate Cutting-Force Measurement with Smart Tool Holder in Lathe

**DOI:** 10.3390/s23094419

**Published:** 2023-04-30

**Authors:** Wandong Song, Jingjie Zhang, Guangchun Xiao, Mingdong Yi, Zhaoqiang Chen, Li Wang, Jun Chen, Chonghai Xu

**Affiliations:** School of Mechanical Engineering, Qilu University of Technology (Shandong Academy of Sciences), Jinan 250353, China

**Keywords:** cutting force, tool holder, semiconductor strain gauge, temperature compensation

## Abstract

Cutting force in lathe work is closely related to tool wear and affects the turning quality. Direct measurement of the cutting force by measuring the strain of the tool holder is challenging because the tool holder design aims to be highly rigid in order to undertake large cutting forces. Accordingly, the most popular dynamometer designs modify the standard tool holder by decreasing the structural rigidity of the holder, which reduces the machining precision and is not widely accepted. In order to solve the issue of the low stiffness of the dynamometer reducing the machining precision, in this paper, the ultra-low strain on the tool holder was successfully detected by the highly sensitive semiconductor strain gauges (SCSG) adjacent to the blade cutting insert. However, the cutting process would generate much heat, which increases the force measuring area temperature of the tool holder by about 30 °C. As a result, the readout drifted significantly with the temperature changes due to the high temperature coefficient of SCSG. To solve this problem, the temperature on the tool holder was monitored and a BP neural network was proposed to compensate for temperature drift errors. Our methods improved the sensitivity (1.14 × 10^−2^ mV/N) and the average relative error of the BP neural network prediction (≤1.48%) while maintaining the original stiffness of the tool holder. The smart tool holder developed possesses high natural frequency (≥6 kHz), it is very suitable for dynamic cutting-force measurement. The cutting experiment data in the lathe work show comparable performance with the traditional dynamometers and the resolution of the smart tool holder is 2 N (0.25% of total range).

## 1. Introduction Precision

Machining/subtractive manufacturing has many advantages over the conventional manufacturing method. Laser-based manufacturing is a widely used, noncontact, advanced manufacturing technique, which can be applied to a very wide range of materials, with particular emphasis on metals [1]. Machining requires online monitoring of the cutting force during the manufacturing processes [2,3,4]. In lathe work, the cutting force is closely related to the tool wear and affects the turning quality. Much effort has been spent on cutting-force measurement in recent decades [5,6], including through piezoelectric sensors, SAW strain sensors, capacitive sensors, FBG sensors, strain-gauge sensors, MEMS sensors, etc. [7,8,9].

Piezoelectric sensors can only measure the dynamic cutting force. The widely used piezoelectric sensor materials include piezoelectric ceramics and PVDF [10]. Due to the compact size, a piezoelectric sensor array was embedded in the tool shank for three-component cutting forces in XYZ [11]. High sensitivity enables piezoelectric ceramic force sensors with a flexible hinge to be mounted on FTS for micro/nano cutting measurements in XYZ axes [12]. New PVDF piezoelectric film sensors with smaller sizes, organic piezoelectric-material force sensors, have been developed. Wang et al. measured the cutting force of the inserts with different parameters by mounting two piezoelectric film sensors directly underneath the insert on the tool holder [13]. The PVDF piezoelectric film sensor has thin thickness [14]; therefore, Luo et al. embedded PVDF piezoelectric film sensors underneath every insert of the milling cutter system. The processing effect shows that each can collect the force data of a single insert for analyzing the failure mechanism of milling inserts [15]. Although there are many advantages of using piezoelectric force measurement, it is limited by the high cost of piezoelectric sensors and their inability to measure static force. On some occasions, the resistance strain gauge force measurement effect is better. 

In order to achieve higher accuracy and a more reliable measurement, the SAW-based smart cutting tool proposed by Chen et al. enables reliable measurement of cutting forces in ultra-precision machining with cutting depths from 0.1 mm to 0.8 mm, and it is in close agreement with dynamometers on the main cutting-force measurement [16]. Using the SAW strain sensor as a sensing unit mounted on the tool holder, Wang et al. achieved constant cutting-force machining of difficult-to-machine materials such as titanium alloys [17]. Capacitive sensors and FBG sensors have also been tested to measure cutting force. Xie et al. modified a standard commercial tool holder to make it the force-sensing element, then six capacitive sensors and other electronics were incorporated into the tool holder as a whole system. However, its relative error was close to 10% compared to a commercial table dynamometer [18]. In addition, Huang et al. proposed a smart tool that has little effect on the structure and characteristics of machine tools due to its small size and light weight. Fiber Bragg grating (FBG) sensors enable the measurement of triaxial cutting-force components in harsh machining environments [19]. The SAW strain sensor and FBG strain sensor have very high accuracy but low economy.

The sensitivity of the semiconductor sensor is improved to a new level with the progress of material science. Zhao You et al. designed a high-precision dynamic cutting-force self-sensing intelligent tool, using the tool holder as an elastic sensitive element, designing a groove structure on the surface of the tool holder, and encapsulating a semiconductor strain gauge with a piezoresistive effect in the groove. The static calibration sensitivity and general accuracy of the main cutting-force direction are 0.1 mV/N and 3.35%, respectively, and those of the feed force direction are 0.1 mV/N and 3.27%, respectively; the lateral cross-interference error is within 3% [20]. However, the smart tool completed in this way weakens most of the rigidity of the holder [21,22,23], which will seriously affect the machining accuracy and measurement range. The Kistler dynamometer uses piezoelectric ceramic sensors, which are limited by the principle of piezoelectric sensing. Piezoelectric ceramics cannot accurately measure a constant force. Strain sensors are particularly suitable for measuring low-frequency signals, such as cutting forces in lathes. In addition, there is a scheme to estimate the cutting force through the power consumption of the machine tool, which is used in cutting where additional equipment cannot be installed for measurement [24]. Prof. Luis presented a data acquisition system that simultaneously allows the recording of cutting forces and the cutting tool position, which can be used for the analysis of the processed workpiece [25]. There is a methodology for the assessment of the geometrical accuracy of a multi-axis machine called a multitasking machine, which comments on the importance of stiffness in machining [26].

It can be found from comparisons of numerous measurement solutions that the piezoelectric sensor has the advantage of high sensitivity, but it can only measure the dynamic cutting force [27]. There is a low economy of the SAW sensor and FBG sensor in measuring cutting force. Semiconductor strain-gauge sensors’ direct measurement of the cutting force by measuring the strain of the tool holder is challenging because the tool holder design aims to be highly rigid in order to undertake large cutting forces. The temperature error caused by its sensitivity to ambient temperatures seriously affects the measurement accuracy [28]; only according to the actual-use environment can performing temperature compensation meet the basic requirements for accurate signal measurement. In summary, the target of the smart tool holder developed in this paper is to be small in size and solve the problem of keeping original stiffness and temperature compensation error.

## 2. System Design 

### 2.1. Device Design

With the continuous development of micro-electromechanical systems, various technical solutions and sensors for cutting-force measurement have been proposed in response to the measurement requirements of cutting-force signals used in tool state monitoring in cutting [12,29]. Compared with other various force measurement technology solutions, the resistance-strain type force-measurement technology has a more reliable cutting-force detection system performance, a more mature technical solution, and a lower application threshold. The smart tool holder proposed in this article is based on the standard turning tool holder. The integrated highly sensitive semiconductor strain gauges (SCSG) detect the ultra-low strain on the surface of the tool holder. The SCSG signal is compensated by the BP neural network algorithm to obtain a more accurate main cutting force, and the main cutting force is indirectly measured successfully. Because the turning tool holder moves continuously with the machining surface during turning, the strain-gauge sensor unit is directly integrated with the turning tool holder, and the related signal acquisition and processing circuit is placed outside the cutting area and connected to the sensing unit by wires [30].

**Figure 1:**
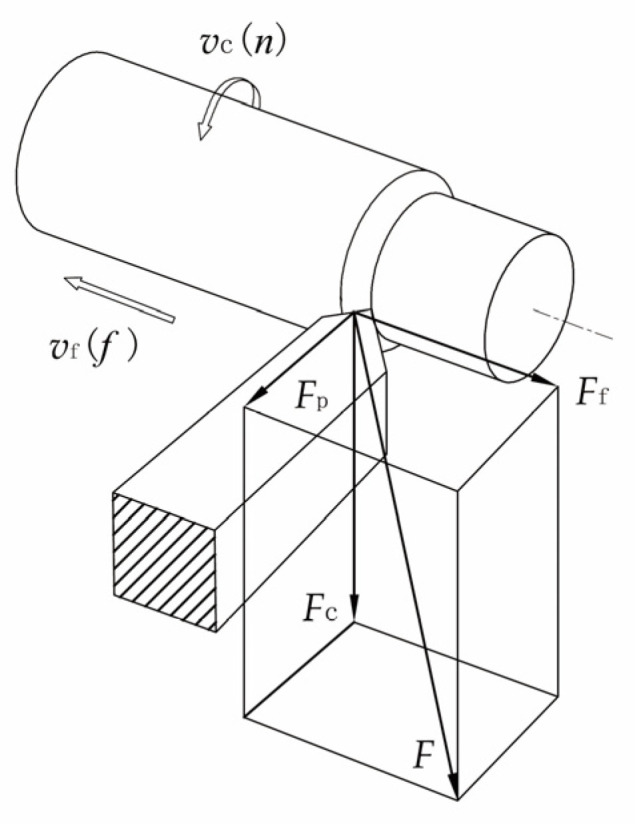
The components of cutting force.

The working principle of the semiconductor strain gauge is based on the strain resistance effect. Figure 1 shows that the cutting force is decomposed into three directions: main cutting force (Fc), feed force (Ff), and back force (Fp). Fc is the most energy-consuming component in turning, and it is also one of the most important signals for monitoring the turning state. The turning tool holder strain caused by the main cutting force is the worthiest of measurement and research. On the one hand, the outstanding advantage of the semiconductor strain gauge is its high sensitivity coefficient, which is 50 to 80 times higher than that of the metal strain gauge; on the other hand, its disadvantages are its serious output nonlinearity and large temperature coefficient, which require the use of temperature compensation technology to improve the performance of the semiconductor strain gauge. In this article, a high-sensitivity semiconductor strain gauge is used to realize the main cutting-force measurement function, and the factors that cause temperature errors are analyzed; to place the NTC thermistor temperature probe close to the semiconductor strain gauge and stick it in the force-sensing area to measure temperature changes; and to use hardware compensation combined with the BP neural network software compensation method to achieve the purpose of accurately sensing the main cutting force.

### 2.2. Finite Element Simulation and Force-Sensing Area Design

Cutting tools of traditional lathes are generally composed of a cutting tool holder and a cutting insert fixed on it. In the turning process, the entire tool system, including the turning tool holder, can be equivalent to a cantilever beam structure, and hence, the cutting force F withstood by the turning tool holder can be equivalent to the force applied to the cantilever beam.

A smart tool holder based on a 45-degree external turning tool holder (MSSNR2020K12) with the function of force-sensing was developed in this study. The tool holder is made of 42CrMo steel, and its parameters used in finite element simulation are shown in Table 1. 

**Figure 2:**
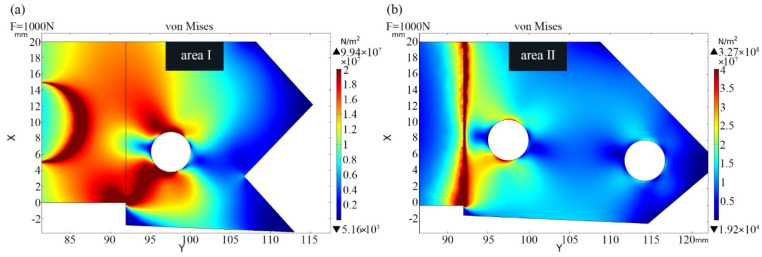
The stress distribution of the tool holder: the stress distribution on the upper (**a**) and lower (**b**) surfaces of the tool holder.

Finite element simulation was performed to study the stress and strain distribution of the tool holder under the action of the main cutting force *F*_c_ [31]. In static stress analysis, the simulated main cutting force is gradually increased from 0N to 1000N. The change of the simulated main cutting force obviously affects the stress on the upper and lower surfaces of the tool holder. The stress distribution on the upper and lower surfaces of the tool holder is shown in Figure 2a,b when F is 1000N. The tensile stress is distributed on the upper surface (a), and the compressive stress is distributed on the lower surface (b). The size of force-sensing area should be larger than the size of integrated strain gauge, and as can be observed from Figure 2a,b, the upper area I and lower area II surfaces of the tool holder meet this demand.

### 2.3. Strain Gauge and Measurement Circuit

The sensitivity of semiconductor strain gauges is about 50–100 times higher than that of metal strain gauges. In this article, semiconductor strain gauges with a silicon strip as substrate were used (SYP-120, Bengbu, China, Jinyu Sensor Co. Ltd.) The performance parameters of the semiconductor strain gauges are shown in Table 2.

**Figure 3:**
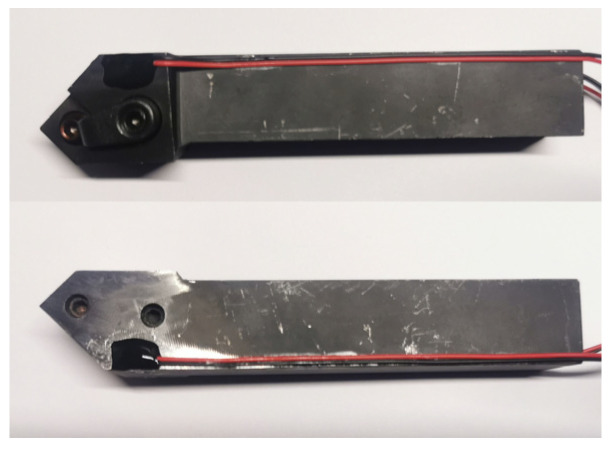
Cutting tool holder with affixed semiconductor strain gauge.

The two selected semiconductor strain gauges were affixed on area I and area II of the cutting tool holder using epoxy resin adhesive, respectively. The sensitive element of the semiconductor strain gauge is extremely vulnerable to external forces, leading to fragmentation damage. Therefore, in addition to operating strictly in accordance with the bonding process, it is also necessary to coat a layer of silicone rubber on the outer layer of the bonded semiconductor strain gauge to prevent moisture and corrosion, as shown in Figure 3. 

**Figure 4:**
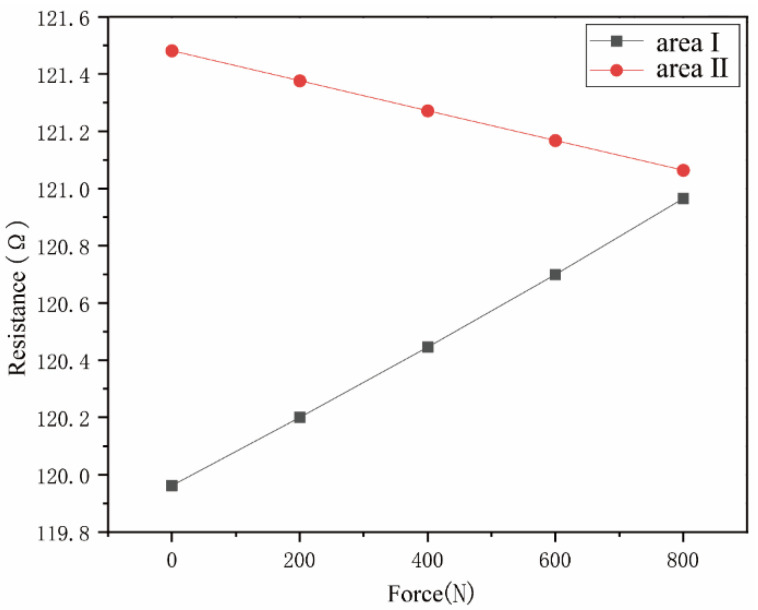
Resistance changes of semiconductor strain gauge.

The resistance-value change curve obtained by the static loading experiment of the two semiconductor strain gauges is shown in Figure 4. During the gradual loading process, the resistance value of the semiconductor strain gauge changes linearly, and the relative change rates of the resistance value of the semiconductor strain gauge in areas I and II are 0.83% and 0.34%. After calculation, the semiconductor strain gauges sensed that the strains of area Ⅰ and area Ⅱ are 69.13 με and 28.74 με, respectively, which match the simulation results.

The relative change rate of the resistance of the semiconductor strain gauge measured in the test is less than 1%, which is too weak for reliable and accurate measurement. Therefore, it is necessary to design a measurement circuit to convert the resistance signal into a voltage signal and perform signal conditioning. This article uses a DC differential bridge to amplify and measure the corresponding strain-gauge signal. It uses two 1.5 V rechargeable batteries to supply power, and a 600 mAh battery can work continuously for at least 48 h.

### 2.4. Error Analysis and Compensation of Sensing System

Paste the semiconductor strain gauge on the force-sensing area of the tool holder and connect it to the DC differential bridge circuit to form a measurement system. When the tool holder is not affected by any external force and when the ambient temperature and tool holder temperature change, the output signal will also change in the measurement work, and the output signal change caused by this temperature seriously interferes with the normal measurement signal, causing a big error. This error caused by the temperature change is called the thermal zero-drift error.

Reasons for thermal zero-drift error of strain gauges:

1. When the temperature coefficient resistance on a strain gauge is large, the nominal resistance value change caused by the temperature change will be obvious.
2. When the linear expansion coefficient of the strain gauge and the tool holder material are different, the additional deformation caused by the change of temperature causes the strain gauge to generate additional resistance.

Therefore, the total resistance change rate of the strain gauge caused by the temperature change is:

(1) $${\left(\frac{\Delta R}{R}\right)}_{t}={\left(\frac{\Delta R}{R}\right)}_{\alpha }+{\left(\frac{\Delta R}{R}\right)}_{\beta }=\left[\alpha +K(\alpha_{1}-\alpha_{2})\right]\Delta t$$

where:

α—The temperature coefficient of the strain gauge;
K—The strain sensitivity coefficient of the strain gauge;
$\alpha_{1}$—The linear expansion coefficient of the tool holder material;
$\alpha_{2}$—The linear expansion coefficient of the sensitive material on the strain gauge;
Δt—The amount of change in ambient temperature.

The temperature-change output relationship of the semiconductor strain gauge can be known from the piezoresistive effect and finite element simulation. In addition, the thermal sensitivity coefficient of semiconductor strain gauges is −0.18 %/°C, and the sensor measurement of thermal sensitivity drift errors also exists. The output error of the sensing system caused by the temperature error of the strain gauge is mainly manifested as the thermal zero drift and the thermal sensitivity drift. Usually, the two kinds of drift errors affect the output signal of the measurement process. Therefore, the deviation caused by the temperature error cannot be ignored in the sensor measurement, and the temperature error problem must be solved.

#### 2.4.1. Thermal Zero-Drift Compensation

**Figure 5:**
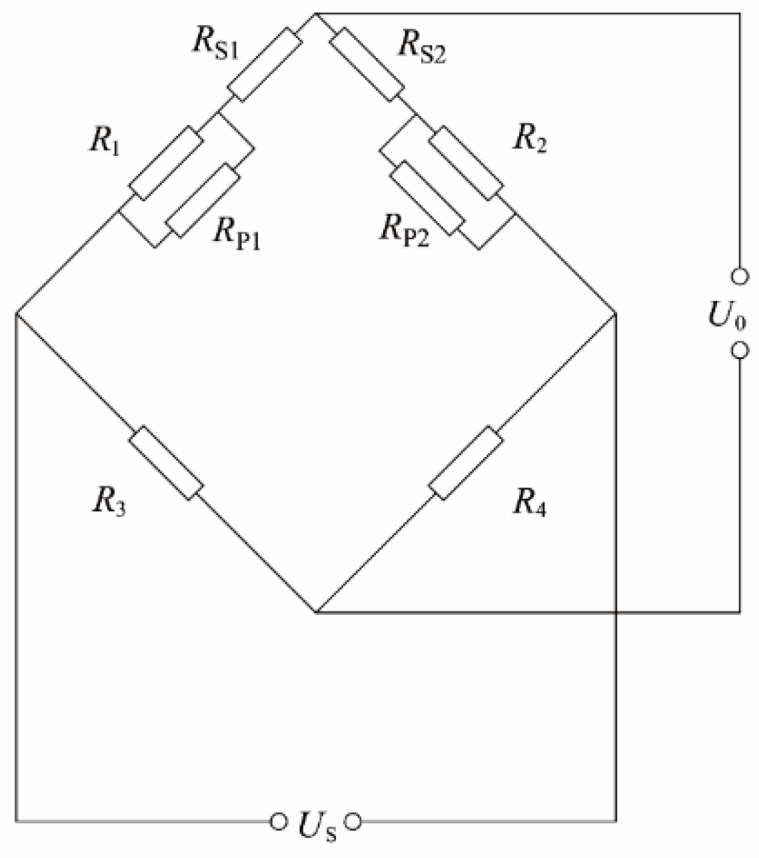
The circuit of series–parallel resistance method.

This article uses a DC differential bridge to amplify and measure the corresponding strain-gauge signal. Therefore, this article chooses to use the series–parallel resistance method to compensate the drift error. In Figure 5, the bridge circuit has four bridge arm resistors, *R*_1_, *R*_2_, *R*_3_, and *R*_4_, among which *R*_1_ and *R*_2_ are sensitive components, and *R*_1_ and *R*_2_ are connected in parallel with *R*_P_ and series *R*_S_ as compensation resistors for zero drift. The function of *R*_P_ is to compensate the resistance change of the strain gauge. Its resistance value is much larger than that of the strain gauge, and it has a small negative temperature coefficient of resistance. When the resistance value of the strain gauge becomes larger due to the increase in temperature, the resistance value of *R*_P_ will drop slightly so that the bridge maintains the previous equilibrium state after the temperature changes. The function of *R*_S_ is zero-setting, and its resistance and temperature coefficient are usually very small.

In this article, the series–parallel resistance method is used to optimize the thermal zero-drift curve of the sensor, and the resistance value of the resistance required for the series–parallel connection is calculated. Before calculation, the temperature curve test of the two semiconductor strain gauges installed on the smart tool holder was carried out, and the resistance temperature curves of *R*_1_ and *R*_2_ were obtained. The series–parallel position of the resistors used for compensation is located on the bridge circuit board, which is affected very little by the cutting temperature of the tool holder. In the calculation, it is assumed that the resistance of the compensation resistor does not change with temperature. *R*_1_ and *R*_2_ are connected in parallel with *R*_P1_ and *R*_P2_, respectively, and only *R*_S_ is connected in series with *R*_1_, and the output expression is:

(2) $$U_{0}=\frac{(\frac{R_{1}R_{P1}}{R_{1}+R_{P1}}+R_{S})U_{S}}{\frac{R_{1}R_{P1}}{R_{1}+R_{P1}}+R_{S}+\frac{R_{2}R_{P2}}{R_{2}+R_{P2}}}$$

where

(3) $$R_{1}=k_{1}T+b_{1}$$

(4) $$R_{2}=k_{2}T+b_{2}$$

(5) $$U_{s}=3$$

Substituting *R*_1_, *R*_2_, and *U*_s_ into Equation (5), *U*_0_ = *f* (*T*), let

(6) $$\left\{\begin{array}{c} A=U_{0}\\ B=\partial U_{0}/\partial T\\ C=\partial^{2}U_{0}/\partial T^{2} \end{array}\right.$$

Taking the working temperature *T* = 35 °C, the temperature coefficient of resistance of the semiconductor strain gauge is calibrated to *k*_1_ = 0.2148; *k*_2_ = 0.2234; *b*_1_ = 116.75 Ω; and *b*_2_ = 117.09 Ω. If you want to obtain a lower zero-point drift output, you need A = B = C = 0. By solving the above equations, the values of series and parallel resistance are *R*_P1_ = 0 Ω, *R*_P2_ = 6301.45 Ω, and *R*_S_ = 1.79 Ω.

After completing the compensation optimization circuit for the sensor’s thermal zero drift and simulating the effect of the temperature of the turning tool holder during the turning process on the sensor’s thermal zero-drift test, the optimization result of thermal zero drift shows that the series–parallel resistance compensation method greatly reduces the thermal zero drift. The maximum output voltage difference before compensation is 7.39 mV, and the maximum output voltage difference after compensation is 0.86 mV, which is a reduction of 88.36%.

#### 2.4.2. BP Neural Network Compensation

Commonly used software compensation methods include the curve fitting method, interpolation method, BP neural network, support vector machine, least squares method, etc. The BP neural network algorithm and several other common software, compared with the compensation algorithm, have stronger nonlinear mapping ability and self-learning and self-adaptive abilities, which can make up for the shortcomings of semiconductor strain-gauge output nonlinearity. This article uses the BP neural network for sensor temperature compensation.

**Figure 6:**
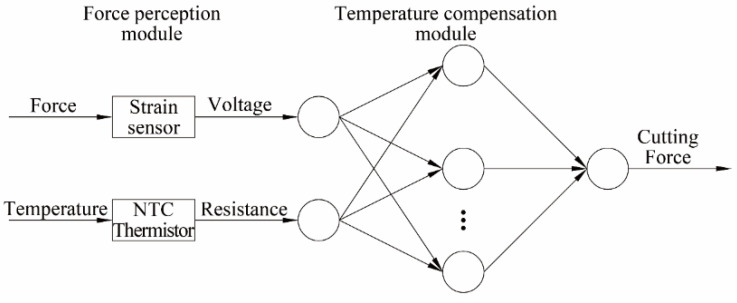
Principle of temperature compensation.

The BP neural network is a kind of error-feedback neural network; its error signal adopts backpropagation, which can realize any nonlinear mapping from input to output. To realize accurate measurement of the main cutting force between the tool and the workpiece, the force-sensing process and the temperature compensation process are required. The principle is shown in Figure 6. The force-sensing process acquires the cutting-force-related signals through a signal acquisition system composed of semiconductor strain gauges and NTC thermistors. An NTC thin film thermistor with a high negative temperature coefficient is stuck close to the semiconductor strain gauge in the force-sensing area. The temperature compensation process uses a BP neural network model to fit the input signals composed of voltage signals and resistance signals that characterize the cutting force. The cutting force is obtained without the influence of temperature error, thereby accurately measuring the main cutting force in the cutting process and providing effective information for monitoring the cutting state.

## 3. Experimental Works

### 3.1. Dynamic Calibration Test

**Figure 7:**
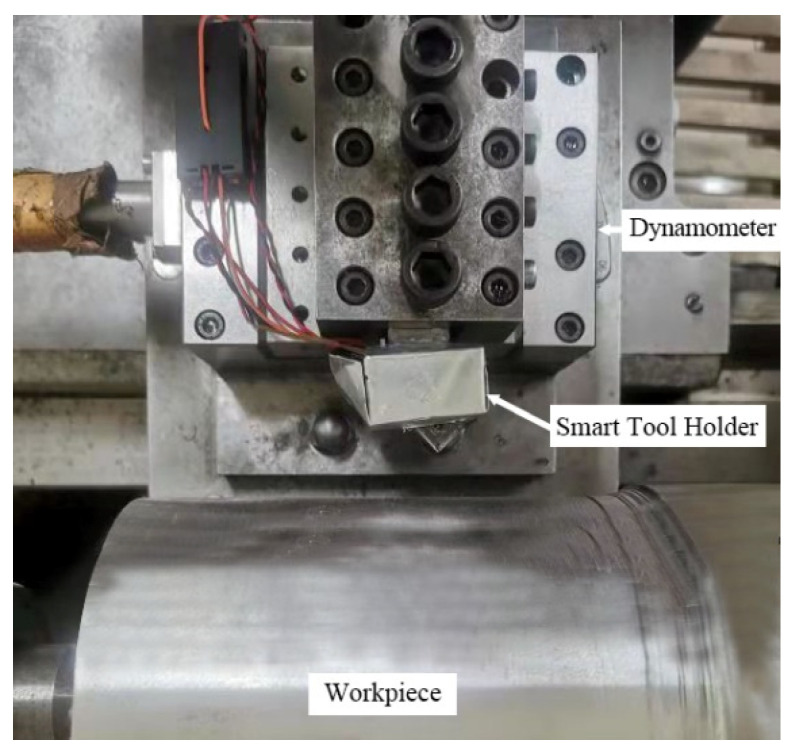
Dynamic calibration experiment.

**Figure 8:**
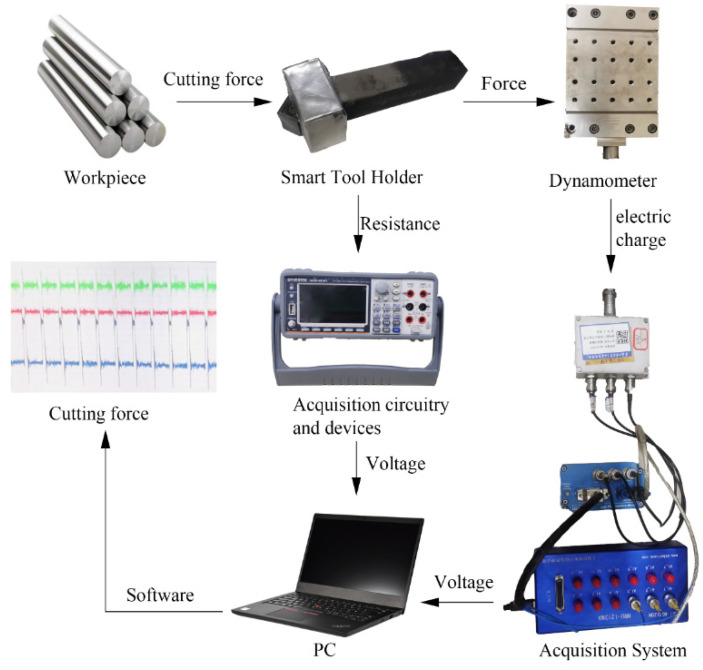
Dynamic calibration experimental signal acquisition system.

A dynamic calibration test was designed, and software compensation methods were used to compensate for the deviation of the output signal of the smart tool holder caused by the temperature gradient during the cutting process. The cutting-force measuring instrument used for dynamic calibration was the 9129AA three-component dynamometer (Kistler, Switzerland). The bandwidth of data acquisition of 9129AA is 50 kHz. As shown in Figure 7, the smart tool holder was installed on the fixture, and the fixture was fixed on the CDE6140A ordinary horizontal lathe tool holder through a dynamometer. The maximum speed of a lathe is 1400 r/min, so the maximum frequency of the cutting-force signal is 24 Hz. In engineering applications, the sampling frequency should be greater than 10 times the signal frequency to avoid obvious amplitude distortion. The workpiece material was 1045 steel after the quenching process. The cutting method was dry turning. The cutting depth varied from 0.1 mm to 0.5 mm with 0.1 mm increments in each pass, with a feed rate of 0.159 mm/rev and a turning speed of 150 m/min. The dynamic calibration experiment signal acquisition system is shown in Figure 8, which is divided into two parts: the smart tool holder signal acquisition system and the 9129AA three-component dynamometer signal acquisition system in this article. The bandwidth of data acquisition of the smart tool holder signal acquisition system is 10 kHz. The signal is transmitted to the computer and processed by the software [27].

The dynamic calibration experiment uses the BP neural network to map the input signal to the cutting-force signal. The input signal is the output voltage signal of the semiconductor strain-gauge sensor measuring the circuit and the resistance signal of the NTC thermistor, and the output signal is the main cutting-force signal measured by the dynamometer. Three channels of signals are synchronized to collect data. This article uses the BP neural network toolbox to train the cutting-force fitting model. The signal collected by the system needs to be input into the BP neural network toolbox after discrete processing. The average value of the main effective and stable cutting-force signal is extracted from the collected signal as the data sample, and the NTC resistance signal and the voltage signal of the semiconductor strain-gauge bridge circuit are also processed in the same way. The whole signal processing flow of the dynamic calibration experiment is shown in Figure 8. 

**Figure 9:**
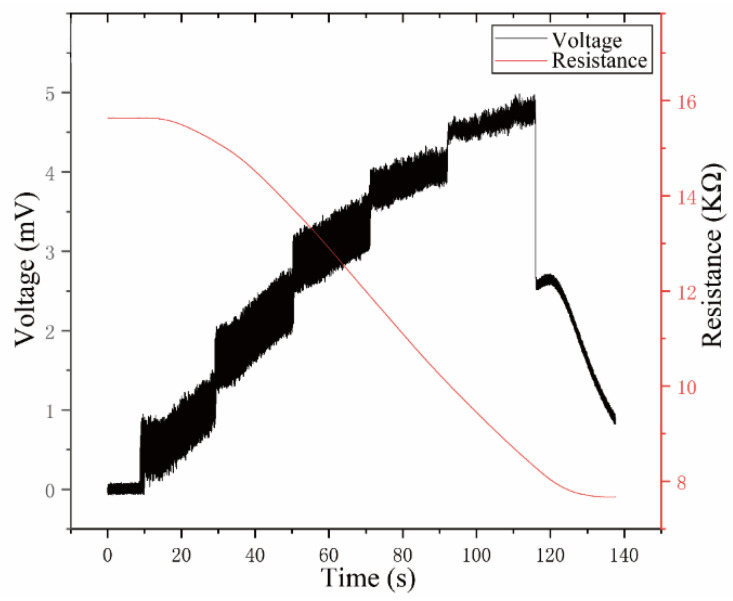
The voltage signal and the resistance signal in the cutting process.

Two sets of data were collected in the cutting experiment. The first set was used to train the BP neural network model, and the second set was used to test the prediction error. The number of samples processed for each set of data was 60,000. The output voltage signal of the smart tool holder and the thermistor resistance change signal are shown in Figure 9. The output voltage signal of the smart tool holder is affected by the cutting temperature. Under the same cutting parameters, as the cutting heat is conducted to the force-sensing area, the voltage signal gradually increases as the resistance of the NTC thermistor decreases. If it is not corrected, the cutting force measured by the smart tool holder is affected by temperature error A with a big deviation. Import the first group of input and output signals into the BP neural network toolbox, and the division ratios of training set, validation set, and test set are 75%, 15%, and 15%.

The three-layer BP neural network was used in this article. The number of hidden layer neurons is six, and each sample signal is mapped from two input signals to one output signal. After training, the model performed 97 iterations, and the optimal model parameters were obtained at the 91st iteration. The mean square error of the optimal model training sample, the verification sample, and the test sample are 1.69, 1.19, and 2.58, respectively. Save the model in the work area for the cutting-force prediction of the second set of data and evaluate the prediction effect of the model.

### 3.2. The Static and Dynamic Characteristics

#### 3.2.1. The Static Characteristics

**Figure 10:**
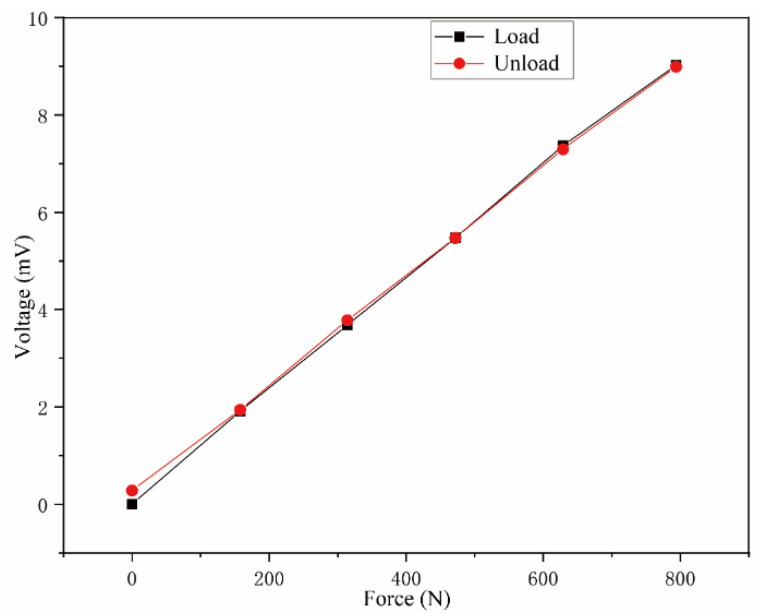
Static calibration curve.

The static calibration test of the smart tool holder developed in this article uses the electronic universal testing machine platform to apply static load to simulate the main cutting force in the machining process to obtain the static characteristics of the smart tool holder. The static calibration curve is shown in Figure 10. The test shows that the smart tool holder developed in this article had a sensitive response to the force exerted on the tool tip. The static sensitivity is 1.14 × 10^−2^ mV/N, which can distinguish the minimum main cutting force of 2.09N. The nonlinear error is 1.34%, and the hysteresis error is 3.16%, achieving a good static force measurement result.

#### 3.2.2. The Dynamic Characteristics

The intrinsic frequency is an important dynamic characteristic parameter of the measuring instrument, which determines the measurement bandwidth of the measurement signal. In this article, the attenuation vibration method is used to measure the first-order intrinsic frequency of the smart tool holder. Ma et al. implemented several impact hammer tests on the “tool holder-spindle system” in papers, and the results were reliable [32]. The intrinsic frequency measurement test is carried out in the working environment of the tool holder, and the measurement process vibration excitation is applied by percussion. In this test, the three-component acceleration tester of Kistler, Switzerland is used to collect the acceleration attenuation signal when the tool holder is excited. An iron rod is used to strike the tip of the tool in the direction of the main cutting force. A total of three percussion excitations are applied, and three acceleration attenuation signals are measured.

**Figure 11:**
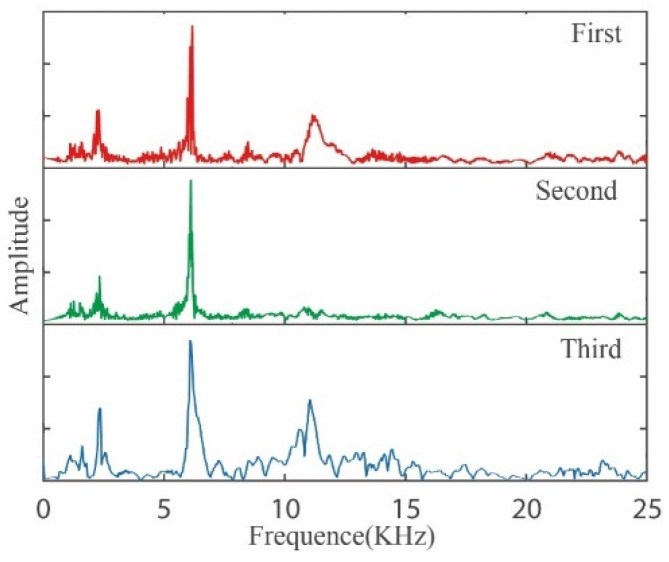
Spectrogram of natural frequency test.

From the measured three-section acceleration attenuation signal spectrum data shown in Figure 11, the intrinsic frequency of the main cutting-force direction of the smart tool holder in this article is 6.08 kHz. The cutting-force signal frequency in turning is the turning frequency of the lathe spindle, so the maximum speed of the lathe spindle for the smart tool holder proposed in this article is 90,000 rpm, and the maximum speed of the lathe is 1400 rpm, so the smart tool holder can meet the requirements of the lathe. Measure the main cutting-force requirements under all machining conditions [33].

### 3.3. BP Neural Network Prediction Results

**Figure 12:**
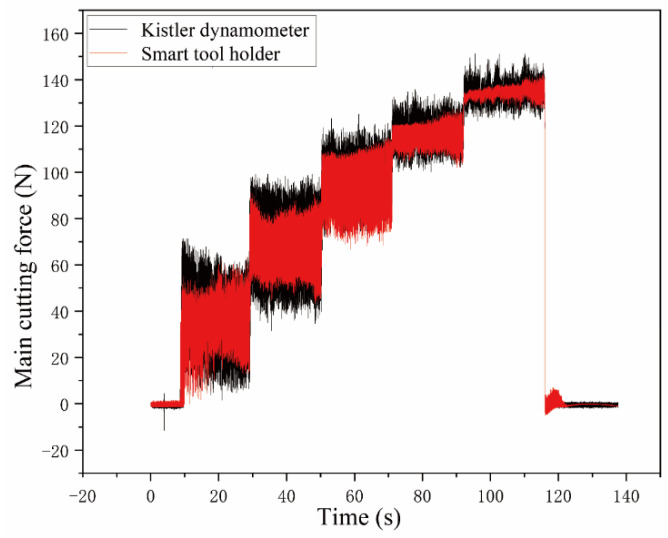
Comparison between the predicted and measured main cutting force.

The trained BP neural network model can be used to compensate the temperature error of the smart tool holder to measure the main cutting force. The model trained using the BP neural network toolbox can be directly called. The two sets of data are used to predict the main cutting force. The comparison with the measurement result of the dynamometer is shown in Figure 12. The result shows that the predicted main cutting force does not increase with the increase of the cutting temperature, and a better temperature compensation effect is obtained. The average cutting force of each cutting depth was calculated for the predicted main cutting force and measured main cutting force. As shown in Table 3, the average relative error of the cutting-force prediction results is 1.48%, which achieves a satisfactory cutting-force measurement effectiveness using smart tool holders.

### 3.4. Performance Results in Cutting Test

After the smart tool holder in this article has been developed and the static and dynamic calibration of cutting-force perception is completed, it is necessary to conduct application performance tests to evaluate the dynamic characteristics of the developed smart tool holder [33]. The application performance cutting test system of the smart tool holder developed in this paper is the same as the instrument used in the dynamic calibration test. The main cutting force measured by the Kistler dynamometer was used as reference data, and the main cutting force was measured and obtained through the trained BP neural network model by the developed the smart tool holder synchronously to evaluate the performance of the developed smart tool holder. 

The performance tests were carried out on a turning test that used CDE6140A ordinary horizontal lathe to turn the cylindrical workpiece. The workpiece material is 1045 steel after the quenching process. The cutting test parameters are shown in Table 4 below. 

**Figure 13:**
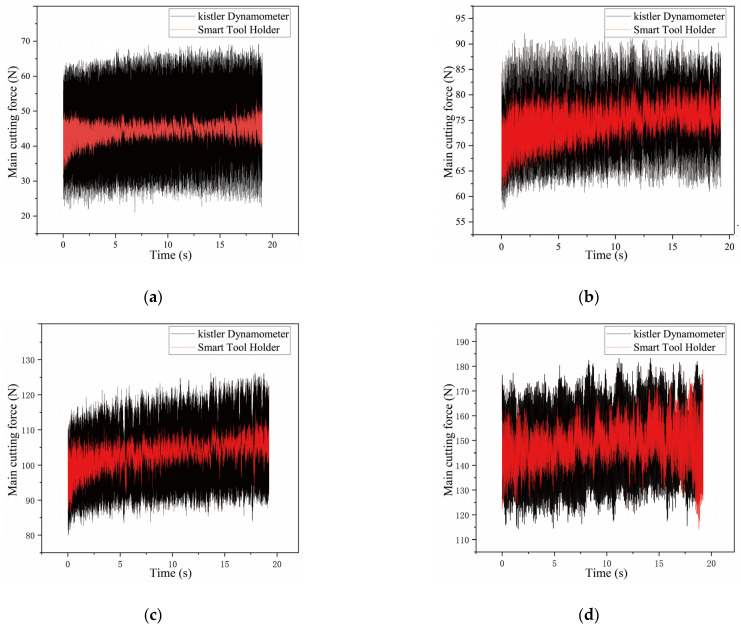

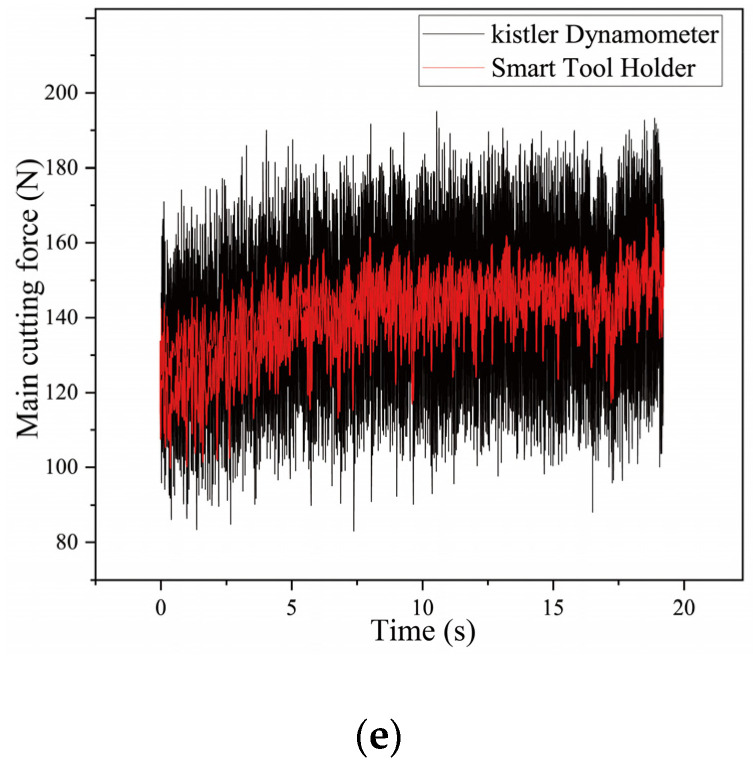
Main cutting-force signal comparison (**a**–**e** are tests 1, 2, 3, 4, 5, respectively).

The main cutting-force signals measured by the smart tool holder and the reference dynamometer are shown in Figure 13. The change amplitude of the main cutting-force signal measured by the smart tool holder and that of the dynamometer signal show an obvious difference. This is because the dynamometer sensor is installed between the tool holder fixture and the lathe tool fixture. The main cutting force is constantly changing during cutting. The inertial force of the tool holder and the tool fixture will act on the piezoelectric sensor of the dynamometer, which induces a larger change amplitude of the charge signal, and then it is collected and transmitted through the charge amplifier. Hence, the amplitude of the main cutting force measured by the dynamometer is larger. The smart tool holder directly measures the stress and strain in the force-sensing area on the tool holder, so the main cutting-force transmission process of the smart tool holder is much simpler than that of the dynamometer, and it responds more sensitively to changes in main cutting force. The stress and strain in the force-sensing area caused by the inertial force are very weak. 

In summary, the smart tool holder is more sensitive to the main cutting force than the dynamometer and has a more accurate response to the main cutting force. The smart tool holder has been dynamically calibrated to complete the compensation of the cutting temperature error, showing a good measurement result. The smart tool holder meets the requirements of obtaining cutting-force information accurately during the cutting process.

## 4. Conclusions

In summary, this paper proposed a smart tool holder for acquiring the main cutting-force signal in the position near the cutting area of the toolholder.

1. The position of the force-sensing area was arranged reasonably by FEA simulation and analysis of the static stress and strain distribution on the surface of the turning tool holder. The semiconductor strain gauges and NTC thermistors were attached to the force-sensing area to detect the strain and temperature.
2. The static calibration results show that the main cutting-force sensitivity of the smart tool holder is 1.14 × 10^−2^ mV/N, the resolution is 2.09 N, and the nonlinear error is 1.34%. The dynamic calibration test of the smart tool holder was carried out. The first-order intrinsic frequency of the smart tool holder mounted on the lathe ≥6 kHz, which is not lower than the original tool holder, indicating that the stiffness of the smart tool holder does not decrease.
3. The BP neural network was trained to compensate the temperature error based on the software compensation, and the average relative error of the prediction result is 1.48%. Finally, the cutting tests show a reliable cutting quality of the smart tool holder while successfully monitoring the main cutting-force signal in the turning process.

Our next step is to add a wireless transceiver and wireless power supply to make the device compact and more convenient to integrate with the tool holder. The ultra-low strain measurement of three-component cutting force is also the next research direction. More miniaturized and wireless three-component cutting-force measuring equipment can be more frequently involved in machining condition monitoring and machining data detection to improve the economic benefits of traditional machining.

## Figures and Tables

**Table 1:** Parameters of 42CrMo steel tool holder.

Parameter	Value
Elastic modulus (N/m^2^)	2.12 × 10^11^
Poisson’s ratio (\)	0.28
Thermal expansion coefficient (1/K)	1.2 × 10^5^

**Table 2:** Semiconductor strain gauge parameters.

Model	SYP-120
Nominal resistance value (Ω)	120 ± 5%
Silicon strip size (mm)	4.7 × 0.22 × 0.02
Nominal sensitivity coefficient	120
Temperature coefficient of resistance (%/°C)	0.13
Temperature coefficient of sensitivity (%/°C)	−0.18
Maximum working current (mA)	50
Operating temperature (°C)	−30~+80
Ultimate strain (με)	5000

**Table 3:** Average predicted main cutting force and measured.

Cutting Depth (mm)	Kistler Dynamometer (N)	Smart Tool Holder (N)
0.1	32.20	33.39
0.2	68.21	67.76
0.3	95.58	94.38
0.4	115.46	116.35
0.5	133.21	134.57

**Table 4:** Application performance cutting test parameters.

Test	Cutting Speed (m/min)	Cutting Depth (mm)	Feed Rate (mm/rev)
1	96	0.1	0.159
2	96	0.2	0.159
3	96	0.3	0.159
4	79	0.4	0.159
5	61	0.5	0.159

## Data Availability

The datasets used or analyzed during the current study are available from the corresponding author on reasonable request. The codes used or analyzed during the current study are available from the corresponding author on reasonable request.
